# Universal Vaccines and Vaccine Platforms to Protect against Influenza Viruses in Humans and Agriculture

**DOI:** 10.3389/fmicb.2018.00123

**Published:** 2018-02-06

**Authors:** Daniela S. Rajão, Daniel R. Pérez

**Affiliations:** Department of Population Health, University of Georgia, Athens, GA, United States

**Keywords:** influenza vaccines, universal vaccines, live attenuated vaccines, immune response, vaccine platform, poultry, swine, vectored vaccine

## Abstract

Influenza virus infections pose a significant threat to public health due to annual seasonal epidemics and occasional pandemics. Influenza is also associated with significant economic losses in animal production. The most effective way to prevent influenza infections is through vaccination. Current vaccine programs rely heavily on the vaccine's ability to stimulate neutralizing antibody responses to the hemagglutinin (HA) protein. One of the biggest challenges to an effective vaccination program lies on the fact that influenza viruses are ever-changing, leading to antigenic drift that results in escape from earlier immune responses. Efforts toward overcoming these challenges aim at improving the strength and/or breadth of the immune response. Novel vaccine technologies, the so-called universal vaccines, focus on stimulating better cross-protection against many or all influenza strains. However, vaccine platforms or manufacturing technologies being tested to improve vaccine efficacy are heterogeneous between different species and/or either tailored for epidemic or pandemic influenza. Here, we discuss current vaccines to protect humans and animals against influenza, highlighting challenges faced to effective and uniform novel vaccination strategies and approaches.

## Introduction

Type A (IAV) and type B (IBV) influenza viruses are responsible for yearly epidemics of respiratory disease in humans. IAVs infect a broad range of avian and mammalian species, including humans. IBVs are considered primarily a human pathogen. According to the World Health Organization (WHO), ~300,000 deaths occur annually worldwide associated with seasonal influenza infections (World Health Organization, 2016). In the U.S., the Centers for Disease Control and Prevention (CDC) estimates that seasonal influenza affects at least 9 million people and results in 12,000–56,000 deaths annually (Centers for Disease Control Prevention, 2017d).

Human seasonal influenza infections vary in intensity, from typically mild respiratory disease with dry cough, nasal discharge, rhinitis and pharyngitis, fever, anorexia, and myalgia to occasionally more severe that can lead to secondary bacterial infections and deaths due to pneumonia (Taubenberger and Morens, 2008). Populations with compromised immunity are at the highest risk of severe and potential life threatening disease, such as chronically ill patients, pregnant women, children, and the elderly (Thompson et al., 2006). In general, H3N2 infections are usually associated with more exacerbated disease than infections with H1N1 or IBVs. There is also the inherent zoonotic risk of IAV strains that circulate in avian and mammalian species, some of pandemic concern, which can result in atypical clinical symptoms and much greater burden to otherwise healthy people (Taubenberger and Morens, 2009). Most cases of H5N1 highly pathogenic avian influenza viruses (HPAIV) presented severe respiratory signs, and gastrointestinal symptoms can also be observed such as vomiting, watery diarrhea, and abdominal pain. Most patients infected with avian H7N9 viruses have had severe pneumonia, with fever, coughing and shortness of breath (Centers for Disease Control and Prevention, 2017e).

IAVs are among the most devastating pathogens for swine and poultry productions. Several genetically and antigenically diverse IAV strains are endemic in swine worldwide and continue to cause significant losses to the swine industry usually as a result of reduced weight gain, secondary infections, and sporadic abortion associated with hyperthermia. In pigs, IAV infections present similar characteristics as the disease in humans, with rapid onset of fever, lethargy, loss of appetite, and coughing (Van Reeth et al., 2012). Avian influenza viruses are endemic in large parts of the world, particularly Asia, the Middle East, and parts of Africa (World Health Organization/World Organisation for Animal Health/Food and Agriculture Organization (WHO/OIE/FAO) H5N1 Evolution Working Group, 2014). Outbreaks of HPAIV can have devastating consequences to an affected country, including direct costs related to the high mortality and indirect costs associated with eradication efforts, control and contention, and loss of markets. The latest outbreak of HPAIV in the U.S. Midwest in 2015 cost the country more than 3 billion dollars (Greene, 2015). In birds, mild forms of low pathogenic avian influenza can range from asymptomatic to mild to moderate respiratory clinical signs, with production losses as a result of lack of feed and water consumption, as well as drop in egg production (Swayne et al., 2013). HPAIV outbreaks are manifested by rapid onset of signs such as depression, neurological disorders, necrotic and swollen wattles and combs, and hemorrhage of the shanks (Swayne et al., 2013) with mortality rates reaching 100%, while some birds might die before showing any clinical signs.

Vaccination remains the most efficient and cost-effective means to prevent and control influenza in human and animal populations. Vaccines rely on the effective stimulation of the immune response against the virus, mostly against the surface glycoprotein hemagglutinin (HA), the primary immunogen of influenza viruses. Influenza vaccines were a high priority for the U.S. military since the Spanish influenza pandemic of 1918–1919 when 1 in every 67 soldiers died from influenza-related infections. However, it was not until 1946 that influenza vaccines became available for the general population (www.historyofvaccines.org). Despite many advances in terms of vaccine manufacturing and production, the technology available for influenza vaccines differs little from its origins and continue to face significant shortcomings about availability and/or efficacy. In this review, we will discuss the use of vaccines to prevent and control influenza in animals and humans. We will highlight challenges faced to establish an effective and standardized vaccination program and discuss new approaches that are being tested to address these issues.

## Influenza viruses

**Figure 1:**
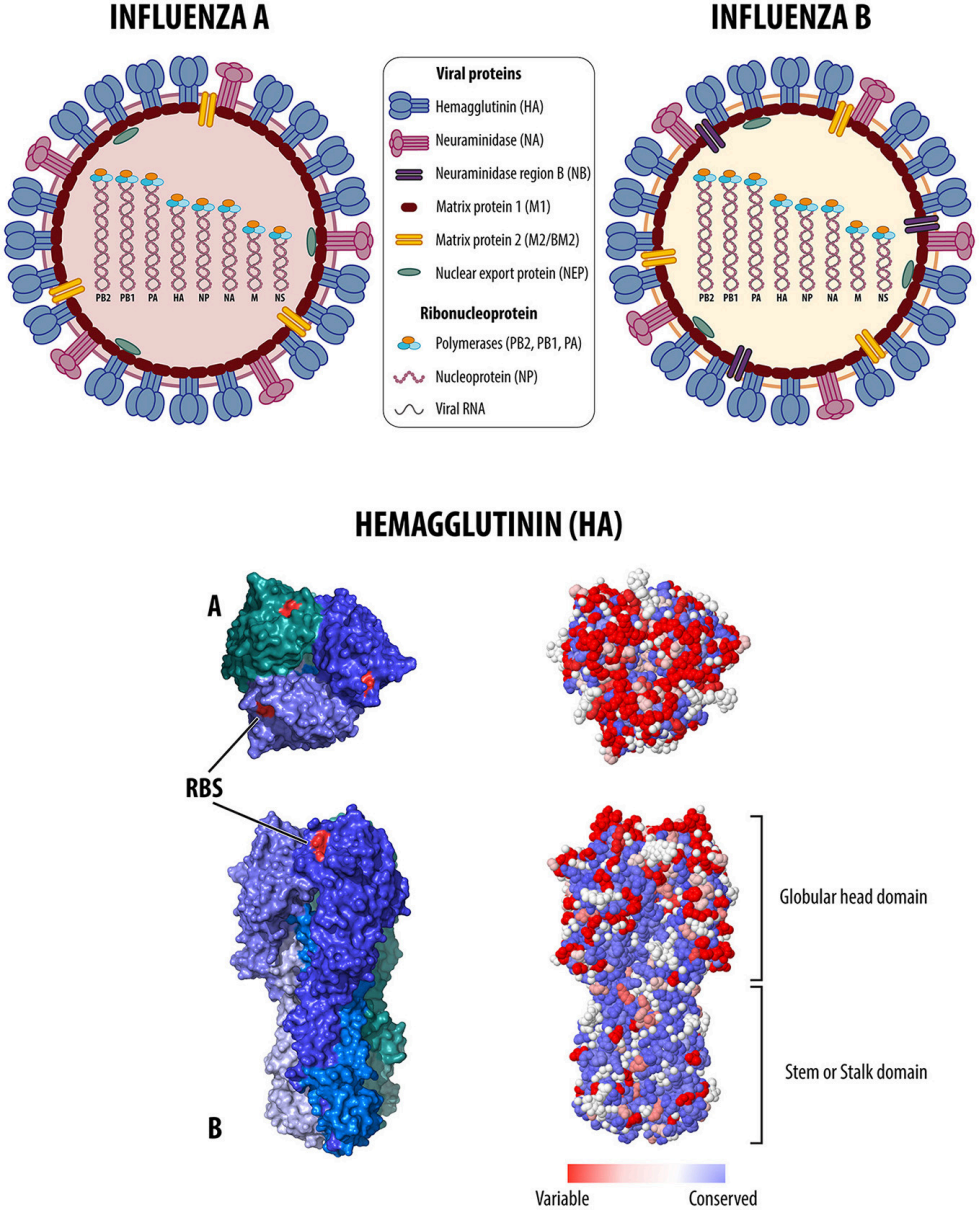
**(Top)** Schematic structure and genome organization of influenza A and B viruses. Haemagglutinin (HA), neuraminidase (NA), matrix protein 2 (M2/BM2), neuraminidase region B (NB) are on the surface of the virus particle. Matrix protein 1 (M1) is associated with the membrane. Ribonucleoprotein complex formed by RNA segments, nucleoprotein (NP) and viral polymerases (PB2, PB1, PA). Non-structural nuclear export protein (NEP). **(Bottom)** The 3D molecular structure of the HA glycoprotein trimer from A/Hong Kong/1/68 (H3N2) (PDB 5T6N), top (A) and side (B) views (structure modified and colored using MacPymol, Schrodinger, LLC). Each monomer has a globular head domain and a stem/stalk domain. On the left panels each monomer is shown with a different color. The receptor binding site (RBS) is highlighted in red. On the right panels, residue conservation for every position in the protein sequence is shown in a color scale, and visualized using the 3D tool available at the Influenza Research Database (www.fludb.org).

IAV and IBV viruses are members of the *Orthomyxoviridae* family. IAV and IBV have in common a genome consisting of 8 single-stranded negative-sense viral RNA (vRNA) segments that encode for at least 10 viral proteins (Knipe et al., 2007; Figure 1). Each vRNA associates with the viral nucleoprotein (NP) and the three polymerase proteins (PB2, PB1, and PA–P-complex), forming the viral ribonucleoprotein complexes (vRNPs). The untranslated regions (UTR) at the 3′ and 5′ end of each segment serve as anchors for the P-complex to carry out transcription and replication in the nucleus of the host cells. The major surface glycoproteins, HA and neuraminidase (NA), plus neuraminidase gene region B (NB) in segment six in IBV, partake in virus attachment and release of progeny virus particles (Knipe et al., 2007). Also on the membrane, the matrix protein 2 (M2 in IAV and BM2 in IBV) acts as a unidirectional proton pump, which is essential early in the infection cycle by allowing the release of vRNPs from the endosome into the cytoplasm and subsequent migration to the nucleus. Matrix protein 1 (M1), NP, and nuclear export protein (NEP/NS2) take part in export of the progeny vRNPs out of the nucleus and assembly into novel virions at the cell surface along with HA, NA, and M2. The non-structural protein NS1 carries out multiple functions during infection with overall antiviral antagonistic activity (Knipe et al., 2007).

The HA is a type I glycoprotein present as homotrimers where each monomer consists of two di-sulfide-linked HA1 and HA2 subunits after cleavage of the HA0 precursor. The HA ectodomain also consists of a globular head domain, and the stalk or stem domain, which are responsible for receptor binding and membrane fusion, respectively (Knipe et al., 2007). The NA is a type 2 glycoprotein present as mushroom-shaped homotetramers. IAV are further classified into H (H1-18) and N (N1-11) subtypes according to the antigenic characteristics of the HA and NA, most of them detected in wild aquatic birds, considered the natural hosts of influenza. Only H1N1 and H3N2 subtypes currently circulate in humans. H1 and H3 subtypes combined with either N1 or N2 subtypes are endemic in pigs. In addition, permanent lineages of IAVs of the H3 subtype circulate in horses and dogs. A wider range of IAV subtypes have become established in land-based birds of the order *Galliformes* (e.g., chickens, quail, turkeys, guinea fowl, among others). Once in *Galliformes*, IAVs of the H5 and H7 subtypes can become HPAIVs due to accumulation of basic amino acids in the cleavage site of the HA0 precursor protein. IBVs are usually restricted to humans, and two antigenically distinct lineages circulate, the Victoria-like and the Yamagata-like lineages.

Influenza A and B viruses accumulate mutations due to the P-complex's lack of proof-reading activity. When these mutations occur in the HA and NA, they lead to antigenic drift, which over time results in escape from earlier immune responses. IAVs can also undergo antigenic shift, whereby a strain with a new HA subtype enters and transmits readily in an immunologically naïve population. Antigenic shift is possible through reassortment resulting from the exchange of gene segments between two or more strains. Reassortment plays a significant role in the evolution of IAVs in the natural reservoir and during the emergence of pandemic strains.

## The animal-human interface of influenza ecology

IAVs that typically infect a particular host species can sometimes cross the species barrier and infect a new host. Animal species such as poultry and swine have significant interactions at the animal-human interface, providing ideal environments for zoonotic transmission events of IAV. These zoonotic transmissions pose a significant threat to public health, not only because of the disease severity and mortality seen in some cases, but also the risk of initiating a pandemic if these viruses become adapted to spread among humans. All human pandemics that happened in the last 100 years originated from viruses with gene segments from animal reservoirs (Taubenberger and Morens, 2009). Humans usually become infected with animal viruses as a result of close contact with a particular animal species. Some of the most prominent poultry-adapted IAVs have been responsible for zoonotic outbreaks and are, therefore, of great public health concern. The WHO considers Asian-origin poultry-adapted IAVs of the H5N1 and H7N9 subtypes among those with the greatest pandemic concern. Since the first detection of H5N1 HPAIV in 1997 and re-emergence as an epidemic in poultry in 2003, 860 human infections have been reported, of which 454 were lethal. Most these cases are the result of direct contact with infected poultry (World Health Organization, 2017). In 2013, a novel H7N9 low pathogenic avian influenza virus (LPAIV) emerged in poultry with the capacity to infect humans. Since then, H7N9 IAVs have been associated with 5 epidemic waves in humans in Mainland China, and the start of a 6th wave reported in October 2017. These H7N9 viruses usually result in severe acute respiratory disease, and until September 2017 had resulted in 1622 laboratory-confirmed human infections and 619 deaths (Food Agriculture Organization of the United Nations, 2017). On the fifth wave, HPAIV forms of the H7N9 were detected also associated with direct contact with infected birds (Iuliano et al., 2017). More importantly, some H7N9 HPAIV strains show signs of more efficient airborne transmission in mammalian animal models (ferrets) (Imai et al., 2017).

Despite the usual mild clinical signs, swine-origin IAVs also represent a real threat to public health due to limited population immunity and potential to become widespread. The 2009 H1N1 pandemic was a result of a swine-origin IAV with a unique combination of gene segments that had not been detected in pigs before, and quickly became globally widespread (Smith et al., 2009). After the 2009 H1N1 pandemic virus (H1N1pdm09) established itself in the human population, it transmitted back to pigs, where it continued to reassort with other IAVs of swine origin. In the U.S., one such reassortant known as variant H3N2 viruses, or vH3N2, containing the M gene from the H1N1pdm09 virus, spread to people resulting in 376 human infections since 2011 (Centers for Disease Control and Prevention, 2017c). Human infections with vH3N2 IAVs are linked with close contact with infected pigs at agricultural fairs (Epperson et al., 2013).

IAVs infect a range of hosts with the potential to jump between multiple species. Interspecies transmission among different animal species and/or from animals to humans or vice versa can have important economic and animal and/or public health consequences; so active surveillance programs are of essence for early detection of IAVs in new hosts. IAVs of swine-origins are often detected in commercial turkeys in the U.S. (Guo et al., 2015) while outbreaks of a H3N8 virus in 2004 and of a H3N2 virus in 2015 in North American dogs originated from IAVs circulating in horses and birds, respectively (Gibbs and Anderson, 2010; Schwartz, 2015). IAV transmission events from humans to swine are frequent and have influenced the diversity of viruses circulating in pigs: some of the most prevalent viral lineages that now circulate in pigs in the United States resulted from reverse zoonotic transmission of human H1N2 and H3N2 viruses to swine (Nelson et al., 2012).

## The current scenario for vaccine production

### Vaccination against influenza

Human influenza vaccines are formulated each year to protect against circulating IAVs and IBVs. The influenza vaccine can protect against influenza viruses that are antigenically the same or related to the viruses in the vaccine. Due to the mutating nature of influenza viruses, annual vaccination is recommended. Originally, these vaccines were available as trivalent inactivated vaccine (TIV) formulations (containing an A/H3N2, an A/H1N1, and an influenza B strain recommended by WHO). However, two antigenically distinct lineages of influenza B viruses have circulated globally since the mid 1980's with limited cross-protection between them (Ambrose and Levin, 2012). Therefore, quadrivalent inactivated vaccine (QIV) formulations are now available, containing an extra influenza B strain representing both lineages (B/Victoria and B/Yamagata). Recommendations for influenza vaccination vary between countries. Most countries recommend vaccination in children, the elderly, chronically ill, and health care professionals. In the U.S., CDC's Advisory Committee on Immunization Practices (ACIP) recommends routine annual influenza vaccination for all persons aged 6 months or older (Grohskopf et al., 2016). The U.S. Flu Vaccine Effectiveness (VE) Network coordinated by the CDC has estimated that the overall, adjusted vaccine effectiveness for influenza seasons from 2005-2016 was between 10 and 60%, with recent studies showing that vaccination reduced the risk of influenza illness by 40–60% when circulating viruses match the vaccine strains (Centers for Disease Control and Prevention, 2017b). However, vaccine efficacy can vary widely between seasons, depending particularly on how well matched are vaccine and circulating strains, and on characteristics of the person being vaccinated. For instance, during the 2014–2015 season, the overall vaccine effectiveness was 19%, and only 6% against the H3N2 component because two thirds of the circulating strains were drifted from the previous year (Zimmerman et al., 2016; Centers for Disease Control and Prevention, 2017b).

Vaccination of pigs is routinely used in several countries, but not all endemic countries vaccinate their herds. In swine, vaccination is typically done in gestating sows to transfer maternally derived antibodies (MDA) through colostrum to their litters, in two to three doses before farrowing (Rajao et al., 2014). Typically, vaccination of sows will protect the litter from clinical signs of disease, and MDAs persist until about 14 weeks of age in piglets (Markowska-Daniel et al., 2011). Occasionally and in herds with high virus circulation, vaccination in growing pigs can protect them against infection once MDA have weaned.

Vaccination is common in some countries where avian influenza viruses have become endemic to prevent and control at-risk populations, most commonly against H5, H7, and H9 viruses, particularly in Egypt, Vietnam, China, Indonesia, and Mexico (Domenech et al., 2009; Spackman and Pantin-Jackwood, 2014). Vaccination has also been successfully used as a control tool to aid in HPAIV eradication programs in poultry and wild birds in some countries, in situations where the regular stamping-out protocols are not enough to control spread and poses a threat to food supply reviewed in Spackman and Pantin-Jackwood (2014). Vaccination of poultry against influenza viruses is still seen with reluctance by many, due to the understanding that vaccines can protect against clinical signs but not infection and, therefore, mask outbreaks and may favor the spread of HPAIV.

### The immune response elicited by vaccination

The local innate immune response is critical for limiting viral replication at the initial phase of infection. Particularly, alveolar macrophages (Kim et al., 2008), natural killer (NK) cells (Gazit et al., 2006), and neutrophils (Tate et al., 2009) have a crucial role in restricting viral spread and reducing disease severity. Infected cells recognize influenza virus RNA via pattern-recognition receptors (PRRs). Activation of PRRs stimulates production of pro-inflammatory cytokines and type I interferons (reviewed in Kreijtz et al., 2011). Inactivated vaccines are poor inducers of innate immunity to give immediate protection. However, a study has demonstrated that binding of UV-inactivated influenza virus to sialic acids (SAs) can trigger intracellular signals for activation of IFN-inducible genes and cytokine production in primary human dendritic cells (Ramos et al., 2011).

Immunization with traditionally inactivated influenza vaccines primarily induces virus-specific adaptive antibody responses. Antibodies against the HA protein, predominantly the globular head domain, are the major protective response against influenza since they usually correlate with surrogate *in vitro* assays such as the hemagglutination inhibition (HI) or virus neutralizing (VN) assays. HI-titers ≥1:40 are considered protective and the accepted standards used by regulatory agencies (Montomoli, 2008). A major hurdle to overcome is that the HA globular head is highly variable and harbors mutations that can lead to antigenic variation. Most HA-head-specific antibodies are only protective against closely antigenically related viruses. In contrast, the stalk domain is more conserved. Influenza infection can result in low levels of antibodies against the stalk region, but these antibodies can have broadly neutralizing activity (Ekiert et al., 2009). In pigs, however, vaccine-induced antibodies to a linear epitope located in the stalk domain increased virus fusion and enhanced respiratory disease, possibly involved with a negative effect (Khurana et al., 2013). However, in mice passive transfer of post-vaccination polyclonal sera resulted in reduced lung virus replication and weight loss compared to animals receiving pre-vaccination sera (Nachbagauer et al., 2014). Further studies are necessary to make clear the levels of HA stem-specific antibodies that correlate with clinical protections and/or if they can negatively influence clinical outcome. Antibodies against the other surface proteins, NA and M2, are not considered neutralizing by traditional assays but studies showed that they limit virus replication and spread *in vivo*.

The CD4+ T lymphocytes and CD8+ T cytotoxic lymphocytes (CTLs) mediate the cellular arm of the adaptive immune response against influenza and play an important role in viral clearance and cross-protective immunity. Virus-specific CD8+ T cells recognize influenza peptides presented by major histocompatibility complex class I (MHC-I) molecules present on the surface of infected cells, resulting in cell lysis. These CTLs are mainly directed against the relatively conserved internal proteins NP, M1, and the P-complex subunits, which confer a high degree of cross-reactive immune response to various influenza strains in humans and swine (Heinen et al., 2001; Hillaire et al., 2013). Not only these CTL aid in virus clearance, but also with protection against disease severity. CD8+ T cell activity correlated with lack of virus shedding, decreased risk of fever, and reduced influenza-like illness in people infected with the H1N1pdm09 virus (Sridhar et al., 2013). After infection in humans, memory CD8+ cell populations are found in lymphoid organs, in circulation or residing in the lungs, are long-lived, and can act upon subsequent infection (van de Sandt et al., 2015). In swine, memory T cells are CD4+/CD8+ double-positive, of which numbers increase in respiratory lymphoid organs and lungs after infection (Khatri et al., 2010). Antigen-presenting cells, such as dendritic cells (DCs), macrophages, and B cells, present peptide fragments of processed antigens on their surfaces in the context of MHC class II (MHC-II) molecules and activate CD4+ T cells. CD4+ T cells are predominantly helper cells, and offer co-stimulatory signals for the priming of B cells (Alam et al., 2014) and CD8+ T cells (Schoenberger et al., 1998) after. Pre-existing CD4+ T cells were also associated with disease protection and lower virus shedding during influenza infection (Wilkinson et al., 2012). Current traditional inactivated vaccines do not stimulate cellular immune responses and, therefore, are usually less effective against heterologous influenza infections, but novel technologies to stimulate T cell immunity are being tested (Soema et al., 2015b) and are discussed below.

### Seasonal vaccine strain selection and production

Identifying circulating viruses, their spatial/temporal trends, and their antigenic relationships to other contemporary and vaccines strains are crucial for an effective influenza vaccine. Antigenic drift requires frequent vaccine updates to match circulating viruses and meet efficient coverage. The WHO's Global Influenza Surveillance and Response System (GISRS) monitors seasonal viruses' evolution by using data obtained year-round from more than 130 national influenza centers located in more than 100 collaborating countries. Meetings are held twice a year to select vaccine strains for the following influenza season. The meetings take place in February for the Northern Hemisphere and in September for the Southern Hemisphere, at least 6 months before their respective influenza seasons. Based on antigenic data from ferret antisera, paired serologic analysis of human samples, and virologic surveillance data, the GISRS recommends that vaccine strains are either maintained or updated to offer more effective protection. Usually following WHO recommendations, each country and its regulatory agencies decide about the influenza vaccines licensed in their territory. In general, each vaccine strain in the vaccine requires an update every 2–3 years, with at least one strain updated each year.

### Vaccine strains selection and production for animal use

Except for vaccine strain selection against equine influenza, for systematic vaccine strain selection or updates is lacking for other animal production systems. A panel of specialists coordinated by the World Organization for Animal Health (OIE) meets annually to analyze surveillance and epidemiological data related to worldwide circulation of IAVs in horses. Genetic sequences and antigenic data are used for recommendations of suitable vaccine strains for inclusion in commercial equine influenza vaccines (Paillot, 2014).

The U.S. Department of Agriculture (USDA) established a national swine IAV surveillance system in 2009. Similarly, efforts are also being made to intensify surveillance in European countries through the European Surveillance Network for Influenza in Pigs (ESNIP) (Anderson et al., 2013; Simon et al., 2014). Both of these efforts have generated consistent flow of genetic data that is useful for vaccine strain choice. Still lacking are collaborative panels to continuously analyze this surveillance data compared to antigenic characteristics of main circulating strains. Unlike IAVs in humans, IAVs in pigs do not follow common evolutionary trends and tend to adopt unique evolutionary pathways depending on the country, region, and even at the farm level. The high diversity and heterogeneous distribution of different viral lineages circulating in pigs represents a major obstacle for effective vaccine choices against swine influenza (Anderson et al., 2016; Lewis et al., 2016). Therefore, swine influenza vaccine manufacturers make independent decisions about the strains to include in their products, and those formulations are not often updated. Likewise, despite intense routine worldwide IAV surveillance in intensive poultry producing areas, the lack of structures to better assess the effects of the use of vaccines in the perpetuation and evolution of IAV in poultry often means that the available commercial vaccines available are not antigenically matched, resulting in less-than-optimal protection (Spackman and Pantin-Jackwood, 2014).

More recently, the OIE along with the Food and Agriculture Organization of the United Nations (FAO) launched a worldwide collaborative network of experts on animal influenza (OFFLU) to support the veterinary community to better manage and reduce the risk of IAVs of animal origin to animal and public health (http://www.offlu.net). This network aims to help recognized emerging strains, provide training and advice, to increase collaboration and data sharing within the scientific community, and to help promote animal influenza research and development.

## Conventional vaccines against influenza

Most influenza vaccines on the market for humans and animals are inactivated influenza vaccines (IIV). For humans and horses there are also live-attenuated influenza vaccines (LAIV) available (Table 1). Regardless of the vaccine strain or manufacturing platform in which the vaccine is made, influenza vaccines are typically produced by growing the target viruses in chicken eggs, which is reliant on continual egg supply. For the most part, seed strains used for human vaccines are typically reassortants generated by the old-fashioned, classical co-infection method to get the target virus HA and NA gene segments in the background of six gene segments derived from an egg-adapted high growth master donor strain, usually A/Puerto Rico/8/34 (PR8) for IAVs and B/Lee/4/40 (B/Lee) for IBVs. Unfortunately, most recent influenza viruses do not grow well in eggs which affects the efficiency of the traditional reassortment method. More importantly, forcing these vaccine viruses to grow in eggs often results in egg-adapted changes associated with antigenic mismatches. In the case of HPAIVs, their inherent high lethality for chicken embryos makes them unsuitable as vaccine strains. Some of these limitations are overcome by reverse genetics techniques that allow generation of whole recombinant influenza viruses entirely from cloned DNA (Neumann et al., 1999; Hoffmann et al., 2000). Thus, reverse genetics allowed to increase the yield of some of the components of LAIV or to mutate the cleavage site of H5N1 HPAIVs and make them suitable for preparation of vaccine seed stocks for pandemic preparedness or as poultry vaccines. Whole wild-type viruses are predominantly used for traditional animal vaccines.

**Table 1:** Summary of experimental and licensed vaccines against influenza viruses for humans and animals described in this review.

**Vaccine platform**	**Vaccine type**	**Species**	**Efficacy**	**Status**	**References**
Inactivated Influenza Virus (IIV)	Whole virus adjuvanted	Poultry, swine, equine, humans	Safe and immunogenic, homologous protection	Licensed	Swayne et al., 2011; Sandbulte et al., 2015
	Split, subunit	Humans	Safe and immunogenic, homologous protection	Licensed	Neurath et al., 1971; Laver and Webster, 1976; Beyer et al., 2011
	Virosomal	Humans	Safe and immunogenic, homologous protection	Licensed	Holm and Goa, 1999; Herzog et al., 2009
	Oil-in-water MF59 adjuvant	Humans	Safe and immunogenic, homologous protection	Licensed	Vesikari et al., 2009, 2011; Domnich et al., 2017
Live attenuated influenza virus (LAIV)	Cold-adapted	Humans, poultry, swine equine	Safe and immunogenic, homologous and partial heterologous protection	Licensed (human, horses), experimental	Song et al., 2007; Pena et al., 2011; Loving et al., 2013; Alam et al., 2014; Gauger et al., 2014; Santos et al., 2017b
	NS1 truncation	Humans, chicken, swine	Safe and immunogenic, homologous and partial heterologous protection	Experimental	Solorzano et al., 2005; Hai et al., 2008; Richt and Garcia-Sastre, 2009; Kappes et al., 2012; Pica et al., 2012; Shi et al., 2016
	Elastase-dependent	Swine	Safe and immunogenic, homologous and heterologous protection	Experimental	Masic et al., 2010
	Rearranged genome	Humans	Safe and immunogenic, homologous protection	Pre-clinical, experimental	Pena et al., 2013; Nogales et al., 2016; Harding et al., 2017
Viral vector vaccine	Modified vaccinia virus ankara (MVA) – conserved proteins	Humans, chickens	Broad cross-reactive response, partial/total homologous protection and partial cross-protection	Phase 1	Lillie et al., 2012; Boyd et al., 2013; Florek et al., 2014; Hessel et al., 2014; Ducatez et al., 2016
	Adenovirus	Human, chicken, swine	Partial homologous/heterologous protection	Phase 1, Experimental	Wesley et al., 2004; Boyd et al., 2013; Crosby et al., 2017
	Alphavirus	Humans, poultry, swine	Partial/total homologous and partial heterologous protection	Experimental	Vander Veen et al., 2012, 2013; Santos et al., 2017a
	Newcastle disease virus	Poultry	Homologous protection	Licensed (chickens), experimental	Liu et al., 2015; Kim et al., 2017
	Herpesvirus Turkey	Poultry	Homologous and partial heterologous protection	Licensed (chickens), experimental	Gardin et al., 2016
Nucleic acid-based	DNA	Humans, chicken, swine	Safe and immunogenic with prime-boost regimen	Phase 1, licensed, experimental	Ledgerwood et al., 2012; Crank et al., 2015; Borggren et al., 2016; Stachyra et al., 2017
	Messenger RNA (mRNA)	Humans, swine	Safe and immunogenic, homologous and heterologous protection	Phase 1, experimental	Petsch et al., 2012; Bahl et al., 2017
Recombinant, Protein-based and Virus-like particle vaccines	Baculovirus expression vector systems (BEVS)	Humans, chicken, swine	Safe and immunogenic, heterologous protection	Experimental	Crawford et al., 1999; King et al., 2009; Baxter et al., 2011; Hernandez et al., 2016
	Virus-like particles (VLPs)	Humans, chicken, swine	Safe and immunogenic, homologous and heterologous protection	Phase 1-2	Low et al., 2014; Pillet et al., 2016; Valero-Pacheco et al., 2016
	VLP-COBRA	Humans	Cross-reactive response, homologous protection	Pre-clinical	Carter et al., 2016
	Headless	Humans	Broadly neutralizing antibody response, homologous and partial heterologous protection	Pre-clinical	Steel et al., 2010; Impagliazzo et al., 2015; Valkenburg et al., 2016
	Sequential immunization	Humans	Broadly neutralizing antibody response, heterologous protection	Pre-clinical	Krammer et al., 2013; Nachbagauer et al., 2014; Kirchenbaum et al., 2015; Ermler et al., 2017
	NA-based	Humans, chicken, swine	Cross-reactive response, homologous and partial heterologous protection	Experimental	Sylte et al., 2007; Van Reeth et al., 2009; Wohlbold et al., 2015
	M2e-based	Humans, swine	Broad cross-reactive response, heterologous cross-protection	Pre-clinical, phase 1, experimental	Turley et al., 2011; Lee et al., 2015; Kolpe et al., 2017; Tang et al., 2017; Tao et al., 2017

### Inactivated vaccines

Inactivated vaccines are the most commonly used products for influenza prevention due to the large history of manufacturing practices associated with relatively low production costs, safety, and effectiveness. There are four types of inactivated vaccines: whole inactivated virus vaccines (WIV), split virus vaccines, subunit vaccines, and virosomal influenza vaccines (reviewed in Soema et al., 2015a; Table 1). Typically, whole-virus killed vaccines produced in 9-11-day-old pathogen-free embryonated chicken eggs, chemically inactivated with formaldehyde or β-propiolactone, concentrated and purified. In split virus vaccines, the virus envelope undergoes disruption by diethyl ether or detergent treatment to expose all viral proteins (Neurath et al., 1971). Subunit vaccines have added purification steps to separate the nucleocapsid and lipids from the surface proteins HA and NA (Laver and Webster, 1976). Despite the loss in viral organization and some loss in immunogenicity, split virus and subunit vaccines are more commonly used against seasonal influenza in humans due to their reduced reactogenicity in comparison with the whole-virus products (Beyer et al., 2011). Virosomal vaccine formulations are mostly used in Europe and consist of unilamellar phospholipid bilayer vesicles carrying surface HA, NA glycoproteins that allow fusion to target cells (Holm and Goa, 1999; Herzog et al., 2009). Inactivated vaccines are usually protective against antigenically closely related strains, but small antigenic changes lead to potential loss of cross-reaction in the HI assay and associated with loss in protection, hence the continuous monitoring for antigenic evolution and updating of vaccine strains.

Most human seasonal TIV/QIV vaccines are available as single dose vaccines for people ≥9 years of age administered via an intra muscular (i.m.) injection, although intradermal (i.d.) formulations are also available. Current guidelines recommend that children 6 months old to <9 years old receive an extra dose administered 4 weeks after the first vaccination. Efficacy of existing vaccines is highly dependent on the age group, with much lower efficacy in children, the elderly, and adults with underlying conditions.

Most swine and poultry influenza vaccines used in the field are inactivated whole virus products combined with potent oil-in-water adjuvants delivered by the i.m. route (Swayne et al., 2011; Sandbulte et al., 2015). Inactivation follows the same techniques as for human vaccines, using formalin, β-propiolactone or binary ethylenimine. The adjuvanted WIV vaccines usually stimulate robust antibody responses against the HA of antigenically similar strains. Just like in humans, circulation of antigenic different variants can decrease vaccine efficacy (Vincent et al., 2008; Grund et al., 2011). Originally, IAV vaccines available for use in swine were bivalent products, but some newer products contain 4–5 strains in response to antigenically distinct viruses circulating within the H1 and H3 subtypes (Sandbulte et al., 2015). Two doses are usually required for vaccination in swine, 2–3 weeks apart (Rajao et al., 2014). In poultry, a single dose is typically effective experimentally but in field conditions multiple doses are commonly applied (Kilany et al., 2010; Kapczynski et al., 2013). Typical large commercial poultry production systems have vaccination programs that include multiple vaccine boosts to protect against avian influenza over the entire production cycle.

### Live-attenuated vaccines

Live-attenuated influenza vaccines (LAIV) are available in several countries for use in humans and horses. Experimental LAIVs are effective in swine and poultry (Table 1). LAIVs have an advantage over inactivated products because they mimic a natural route of infection but with very low rate of adverse reactions. In contrast to inactivated products, vaccination with live products provides both humoral and cell-mediated immunity, and they can induce mucosal IgA responses in the upper respiratory tract, hence providing more comprehensive cross-reactive and longer-lasting immune responses (Loving et al., 2012; Hoft et al., 2017). The major drawback of LAIVs is that they are usually poor stimulators of HI responses compared to inactivated vaccines, hence the HI titer cannot always be used as a correlate of protection for LAIVs. These vaccines are not recommended for use in immunocompromised people because of their inherent risk of developing disease. Other perceived risks associated with the use of LAIVs are vaccine strain reversion and recombination with circulating field strains; however, the different LAIV systems have been consistently safe and stable.

LAIVs for human use were independently obtained by the U.S. and Russia in the 1960's after serial passage in eggs resulting in viruses with cold-adapted, temperature-sensitive mutations that prevented their growth at temperatures higher than 35°C and thus restricting virus replication to the nasal cavity (Maassab et al., 1990). In the U.S., available LAIVs consist of reassortant viruses carrying cold-adapted, temperature sensitive (ts) mutations of the master donor viruses A/Ann Arbor/6/60 for IAV and B/Ann Arbor/1/66 for IBV (Maassab et al., 1990; Carter and Curran, 2011).

An intranasal cold-adapted LAIV produced by MedImmune (FluMist) was licensed in the U.S. in 2003. A quadrivalent version of FluMist received approval in 2012. Overall, adjusted vaccine effectiveness for LAIV against any influenza has been consistent with IIV (~50–60%), showing better effectiveness to some components than others in comparison to IIV (Ambrose et al., 2011; Caspard et al., 2017). After suboptimal protection observed against the H1N1pdm09 component in recent years in children aged 2–17 years (3%), CDC revoked its recommendation for the use of LAIV in the U.S. for the 2015–2016 and 2016–2017 seasons (Centers for Disease Control and Prevention, 2017a). The reasons for the recent lack of protection of the FluMist vaccine is not yet fully understood, and it is not known whether this is a characteristic specific to the H1N1pdm09 strain associated with reduced stability and/or infectivity (O'Donnell et al., 2014) or interference from other components in the vaccine. There is evidence that the LAIV contained a large amount of defective-interfering (DI) viral RNA, which could compromise virus replication in the respiratory tract and thus decrease the immune response to the vaccine (Gould et al., 2017). In contrast, the “Leningrad” LAIV available in Russia since 1987 for children over 3 years of age, adults and the elderly continues to show superior effective protection in children compared to inactivated vaccines (Ghendon et al., 1984; Rudenko et al., 2016). Since 2009, an agreement permitted WHO to grant sub-licenses of the Russian LAIV to manufacturers in developing countries, including China, India, and Thailand (Rudenko et al., 2016).

A modified live vaccine against equine influenza virus (EIV) is the only live-attenuated vaccine approved for use in animals. The vaccine has a cold adapted and temperature sensitive virus derived from the wild-type A/eq/Kentucky/1/91 (H3N8) EIV strain and has been proven safe and efficacious for use in horses in the U.S. (reviewed in Paillot, 2014).

## Current challenges for vaccine manufacturing and effective use

The greatest challenge for vaccine manufacturing for both animal and human influenza is the high variability and rapid evolution of the virus that results on a constant chase for the vaccine virus to match the circulating strains. As a result, current vaccine programs are dependent on extensive surveillance, at a global scale for human vaccines and at a regional/local scale for animal vaccines. Since there are no standardized programs for vaccine selection in the veterinary field, vaccine strains are often mismatched and offer suboptimal protection that can promote, rather than stop, the spread of field strains. Additionally, pigs vaccinated with adjuvanted IIV and then challenged with a heterologous virus of the same subtype can show enhanced respiratory pathology due to strong stimulation of non-neutralizing antibody response, termed vaccine-associated enhanced respiratory disease (VAERD; Gauger et al., 2011). Furthermore, a suboptimal vaccine can result in escape mutants that will pose an increased threat to naïve populations.

Another obstacle for an effective vaccine is the timeline needed for vaccine manufacture and regulations for approval. The schedule for surveillance data collection for human influenza attempts to include the peak of the current season and increase the odds of correctly forecast which viruses are the most likely to circulate during the coming season. However, because manufacturers need to allow enough time for production to account for possible delays, this is not always the case and in some instances the distribution and antigenic make up of circulating viruses at the end of the season differs from the selected vaccine strain, which could result in a mismatch. From strain selection to vaccine distribution takes at least 6 months, without considering possible setbacks such as egg supply obstacles or problems in meeting biosafety and stability requirements. As for animal vaccines, strains are not updated as often and the process to license updated or new products could be even more time-consuming than for human vaccines. Recent changes implemented by the USDA Center for Veterinary Biologics now allow manufacturers to change or replace strains under an existing license without having to go through an entirely new licensing process (Center for Veterinary Biologics, 2007), which presents an advance on the path to advance vaccine programs.

The inherent variation in the immune status of a given population is also a major factor influencing vaccine safety and efficacy. For instance, high-risk groups, such as the elderly or immunocompromised people may not respond optimally to vaccination due to declined immune function (Kunisaki and Janoff, 2009; Lambert et al., 2012). An individual's previous immunity can have a major impact on the vaccine responses. It is well accepted that the first immunological encounter with influenza, either by natural infection or vaccination, will shape up future responses in the life of an individual, a process often called *original antigenic sin* and more recently as *antigenic imprinting*. The resulting secondary exposures tend to boost the antibody response to the priming exposure in total or partial detriment of the immunological response against the new strain, a process known as or *back-boosting* (Fazekas de St and Webster, 1966; Ma et al., 2011; Fonville et al., 2014).

In swine and poultry production systems, maternal derived antibodies in young animals can interfere with the active immune responses to vaccine depending on the age at vaccination (Loeffen et al., 2003; De Vriese et al., 2010). Other obstacles for implementation of vaccine programs in agricultural animals are the costs, the prohibitive withdrawal period post-vaccination, the difficulty to differentiate vaccinated from infected animals and the masking of clinical signs that can result in trade restrictions (Spackman and Pantin-Jackwood, 2014).

## Efforts to avert the setbacks in vaccine use

The limitations mentioned above highlight the need for new technologies and vaccine platforms that could improve vaccine production and availability, but also induce long-lasting broadly protective immunity. Alternative routes of delivery and different vaccination strategies could improve immune response to traditional vaccines. In addition, vaccines that target conserved regions of the virus and result in broader, longer-lasting immune response, termed “universal” vaccines, are also highly desirable and several different technologies developed in recent years show great promise as discussed below (Table 1).

New technologies to improve manufacturing processes, cut production time and costs, and increase production capacity are in demand. Large-scale cell-based production technology provides faster and easier production process, potential fewer issues with cell-adapted virus mutations and reduced risk of allergic reactions to egg-components. Influenza vaccines have been successfully evaluated in several continuous mammalian cell lines, such as Madin Darby canine kidney cells (MDCK), monkey kidney cells (Vero), and human embryonic retinal cells (PER.C6) (reviewed in Milian and Kamen, 2015). Cell-based vaccines produced in MDCK (Flucelvax and Celtura by Novartis) and Vero (Celvapan by Baxter Vaccines) cells received approval for use against human influenza in the US and Europe since 2009 (reviewed in Manini et al., 2017).

### Adjuvants

Adjuvants increase the immunogenicity of vaccines, mainly by improving antigen processing and delivery to antigen-presenting cells and stimulating production of specific immunomodulatory cytokines and innate immune response (de Veer and Meeusen, 2011). Increasing the immunogenicity of vaccines leads to antigen sparing. Adjuvants are not widely used for influenza vaccines in humans, but are particularly desirable to increase vaccine efficacy in the elderly, children, and immunocompromised people. Some antigen formulations, such as peptides and DNA vaccines need adjuvants to improve immunogenicity. A diverse range of compounds serve as adjuvants for influenza vaccines, some licensed (aluminum salt or oil-in-water squalene-based emulsion), others in development (saponins, liposomes, cytokines, polymers) (reviewed in Soema et al., 2015a).

Aluminum salt is the most commonly used vaccine adjuvant for humans; however, it did not show a beneficial effect with the H1N1pdm09 vaccine (Manzoli et al., 2011) or with an avian H5N1 vaccine (Bernstein et al., 2008). The oil-in-water MF59 adjuvant first licensed by Novartis in 1997 in Italy for people ≥65 years of age is now approved in more than 30 countries, including the United States (Centers for Disease Control Prevention, 2016). The MF59-adjuvanted vaccine was highly effective and superior than non-adjuvanted vaccines in the elderly (>65 years) (Domnich et al., 2017). Importantly, M59-adjuvanted TIV is highly immunogenic and induces longer-lasting, broader immune response in children, and increased protection against influenza infection in pediatric populations compared to non-adjuvanted vaccines (Vesikari et al., 2009, 2011). AS03, an oil-in-water adjuvant containing α-tocopherol as an immunostimulant, has been licensed for use with avian-origin H5N1 and H1N1pdm09 split inactivated monovalent vaccines. The AS03-adjuvanted H1N1pdm09 vaccine has been extensively used in many European countries and in Canada; however it has been recently associated with increased number of narcolepsy cases in people <20 years of age (Persson et al., 2017).

In poultry and swine, the population targeted for vaccination is often composed of naïve animals. Most licensed influenza vaccines for use in poultry or swine use oil-based adjuvants to improve the immunogenicity and breadth of the immune response. In chickens, mineral and vegetable oil-based adjuvants (particularly Montanide ISA 70VG and Montanide ISA 71VG, SEPPIC) induced the highest antibody titers compared to other compounds (polymer, mineral nanoparticle, carbohydrate). Interestingly, protection of poultry against a H7N3 strain was not statistically different among various adjuvanted vaccine formulations (Lone et al., 2017).

### Universally protective vaccines

**Figure 2:**
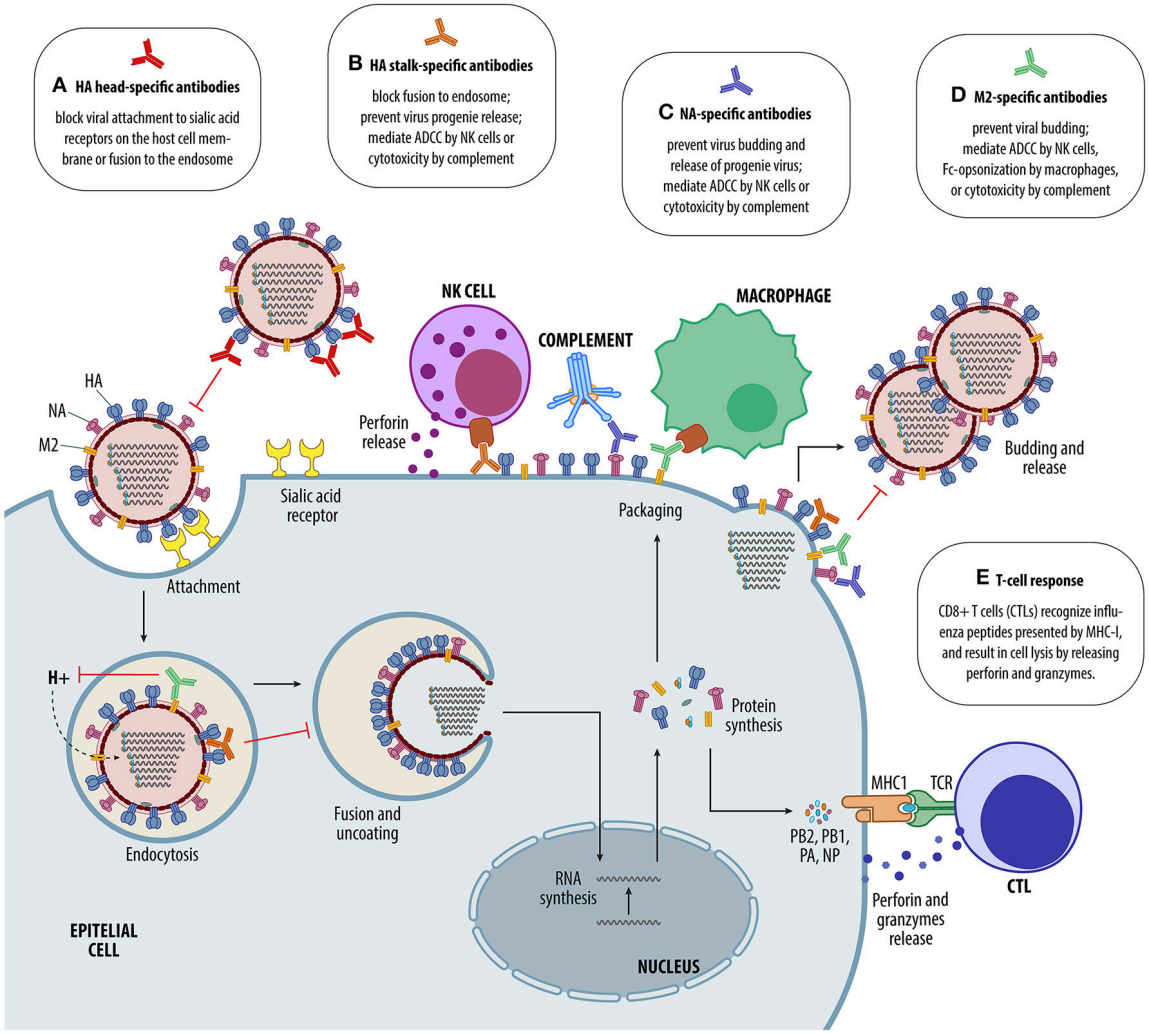
Immune response elicited by different influenza vaccines. **(A)** HA-head-specific antibodies interfere or block virus binding to the sialic acid receptors on the cell surface and prevent virus entry to host cells. **(B)** HA-stalk-specific antibodies prevent virus fusion to the endosome, inhibit budding and release of new virus particles, and mediate antibody dependent cell-mediated cytotoxicity (ADCC) by natural killer (NK) cells or complement activation. **(C)** NA-specific antibodies inhibit budding and release of new virus particles, and mediate ADCC by NK cells or complement activation. **(D)** M2e-specific antibodies inhibit budding and release of new virus particles, and mediate ADCC by NK cells, Fc-opsonization by macrophages or complement activation. **(E)** CD8+ T cells (cytotoxic T lymphocytes, or CTLs) recognize influenza peptides presented by major histocompatibility complex class I (MHC-I) at the surface of infected cells via their T cell receptor (TCR), release cytotoxic granules containing perforin and granzymes, resulting in lysis of infected cells.

New “universal” influenza vaccine approaches attempt to overcome the drawbacks of the highly changing nature of influenza viruses. The objective of these vaccines is to induce cross-protective broadly neutralizing immunity, which depends on stimulating both humoral and cell-mediated arms of the immune system. These “universal” vaccines rely on the concept of developing immune responses against conserved viral epitopes. Most of these strategies show great promise against IAVs and IBVs. The ultimate goal of these strategies is to avoid annual vaccine updates while prolonging the breath of immune responses and decreasing the need for re-vaccination. Broadly protective vaccine candidates that are under investigation target either the highly conserved epitopes of the HA, the NA or the extracellular domain of the M2 protein (M2e) to induce cross-reactive antibodies, and/or target internal proteins like NP and M1 to induce cross-protective T-cell response (Table 1, Figure 2).

Because these novel candidate “universal” vaccines focus on stimulating either T-cell and/or antibody responses against conserved epitopes other than the HA head, their protective responses cannot be evaluated nor quantified by standard methods. Historically, immunogenicity and vaccine protection parameters against influenza rely on the HI assay to measure neutralizing antibodies. An HI titer of 40 correlate with protection in healthy adults corresponding to a 50% reduction in the risk of contracting influenza. In children such risk reduction requires an HI titer of ~110 (Black et al., 2011). Other assays to measure immune responses to influenza viruses are available, such as NA-specific or cell-mediated responses, that could be used as surrogates of protection (McElhaney et al., 2013; Sridhar et al., 2013; Monto et al., 2015). Such assays are not routinely used and need further optimization and standardization. However, they could become extremely useful to predict protection, particularly in populations that show low HI response after vaccination like the elderly or for vaccines that do not elicit HI-antibodies.

#### Universal vaccines targeting HA-specific antibodies

Unlike the head domain, the stalk domain is much more conserved across HA subtypes and phylogenetically classified into two groups: group 1 that includes the H1, H2, H5, H6, H8, H9, H11, H12, H13, and H16 subtypes, and group 2 that includes the H3, H4, H7, H10, H14, and H15 subtypes. Antibodies against the HA globular head neutralize infection by either preventing binding to cellular receptors or membrane fusion (Figure 2A). Stalk-specific antibodies do not usually neutralize the virus (measured by using standard assays), but protect through inhibition of either entry, viral release, by mediating antibody dependent cell-mediated cytotoxicity (ADCC) or complement cytotoxicity (Figure 2B). Anti-stalk antibodies are naturally produced after infection but in low levels compared to those against the immunodominant globular head domain (Corti et al., 2011). Recently, a study found that high levels of HA stalk-reactive antibodies measured by ELISA and high ADCC activity correlated with protection against H1N1pdm09 challenge in mice with passive transfer of sera from H5N1 vaccinees (Jacobsen et al., 2017). Since HI responses against the H5N1 virus were below predictive protective values against the H1N1pdm09, the authors concluded that high levels of HA stalk-specific antibodies were responsible for protection and presented evidence for other parameters associated with correlates of protection.

By using a “headless” HA as antigen, vaccinated mice produced levels of anti-stem broadly neutralizing antibodies (bnAbs) that protected them against lethal homologous (Corti et al., 2011) or heterosubtypic challenge (Steel et al., 2010). The same “headless” HA approach reduced fever after sub-lethal homologous challenge in cynomolgus monkeys (Impagliazzo et al., 2015). In a distinct “headless” approach, mice vaccinated with a conformational polypeptide that mimics the H5 HA stem (mini-stem) resulted in protective responses against lethal challenge with both group 1 (H5 and H1) and group 2 (H3) influenza viruses (Valkenburg et al., 2016). Sequential immunization with chimeric constructs that express the same stalk but irrelevant divergent heads also induced broadly neutralizing, stalk-specific antibodies that protected mice against lethal heterologous challenge (Krammer et al., 2013; Ermler et al., 2017). Anti-stalk antibodies can also be stimulated with sequential immunizations with antigenically different strains within the same group (Nachbagauer et al., 2014; Kirchenbaum et al., 2015).

Interestingly, mice produce cross-reactive neutralizing antibodies directed to the globular head of the HA (Yoshida et al., 2009). More importantly, broadly neutralizing head domain antibodies have been characterized in humans, with activity against a single subtype (e.g., H1 for CH65, H3 for F045-092) or against multiple subtypes (e.g., C05, S139/1) (Whittle et al., 2011; Ekiert et al., 2012; Lee et al., 2012, 2014), suggesting that specific regions of the head domain are targets for “universal” vaccines. Despite the high variability between HA subtypes, attachment to sialic acid receptors on the host cells is a conserved activity (Weis et al., 1988). Antibodies that target highly conserved residues close to or at the receptor-binding site on the HA head result in receptor mimicry and prevent viral-host recognition. Recently, an antibody was shown to result in strong cross-protection against multiple lineages of influenza B in mice and ferrets (Shen et al., 2017).

Another HA-based strategy to develop “universal” vaccines is the Computationally Optimized Broadly Reactive Antigen (COBRA) technology, which uses consensus sequences to design vaccines that represent multiple circulating strains. A COBRA VLP-based H1 vaccine showed broad cross-reactivity to multiple H1 strains and protected mice against H1N1pdm09 challenge either in a monovalent cocktail or in prime-boost regimens (Carter et al., 2016).

#### Universal vaccines targeting NA-specific antibodies

Current influenza vaccines are not standardized for the amount of NA. The stability and correct folding of the NA in these vaccines is not well defined. Antibodies that inhibit NA activity interfere with virus release from the cell surface, reducing the amount of virus progeny produced by the infected cell, and may also mediate ADCC (Figure 2C). The influenza NA protein is antigenically more conserved than the HA protein, and several animal studies have shown that NA-specific antibodies may induce cross-protective immunity against homologous and heterologous strains that are closely related. In one of these recent studies, vaccination of mice with recombinant adjuvanted N1, N2, or IBV NA provided sterilizing immunity against homologous virus and conferred partial protection against heterologous infection; however, heterosubtypic protection was lacking (Wohlbold et al., 2015). Vaccination of chickens with a recombinant N2 with similar homology to the challenge virus and high NI activity protected against mortality but not morbidity (Sylte et al., 2007). When pigs were vaccinated with inactivated adjuvanted vaccine and challenged with a virus containing antigenically mismatched homosubtypic HA and NA proteins, they showed enhanced disease characteristic of VAERD. However, VAERD was abrogated when a matched NA vaccine was used (Rajao et al., 2016).

#### Universal vaccines targeting M2e-specific antibodies

M2 protein is a relatively conserved transmembrane protein of influenza viruses and its extracellular domain (M2e) has been extensively explored as a “universal”antigen candidate for vaccines. Anti-M2e antibodies do not neutralize the virus, instead these antibodies are thought to prevent viral budding, mediate killing of infected cells by NK cells or macrophages through ADCC, Fc-opsonization, or complement activation (El Bakkouri et al., 2011; Figure 2D). M2e is present on the virus' surface in low quantities and, thus, it is poorly immunogenic (Wu et al., 2007). However, strategies to improve immunogenicity, such as adjuvants, multimeric forms of M2e, co-immunization with other influenza vaccines, or fusion with carrier proteins (Kim et al., 2013; Lee et al., 2015; Tang et al., 2017) stimulate broad cross-reactive immune responses and provide protection against heterologous, hetorosubtypic challenge in mice (Lee et al., 2015; Tao et al., 2017). Human clinical trial demonstrated the safety and immunogenicityof different M2e vaccine candidates (reviewed in Turley et al., 2011; Kolpe et al., 2017). Surprisingly, pigs vaccinated with an M2e vaccine and challenged with a heterologous virus showed severe clinical signs compared to control animals (Heinen et al., 2002).

#### Universal vaccines targeting T-cell responses

CD8+ cytotoxic T lymphocytes (CTLs) predominantly target conserved internal influenza virus proteins, such as the NP and M1, and, therefore, generate cross-reactive immune responses to influenza viruses of different subtypes (Gotch et al., 1987; Hillaire et al., 2013; Figure 2E). Vaccines focusing on the induction of CTL responses should provide a broader cross-protective response and are potential candidates for a more “universal” vaccine. The NP and M proteins were shown to contain highly conserved epitopes shared by various subtypes of influenza virus that are recognized by diverse human leukocyte antigen (HLA) backgrounds (Lee et al., 2008), and hence are potential candidates for the development of broadly protective vaccines. A MVA-based vaccine expressing the NP and M1 proteins (MVA-NP+M1) significantly boosted T-cell response in naturally primed vaccinated human subjects and vaccination was shown to reduce clinical disease and virus shedding in intranasally challenged individuals (Lillie et al., 2012). Similarly, chickens primed with an adenovirus expressing a fusion construct of NP + M1 proteins and boosted with an MVA vector vaccine containing the same construct (MVA-NP+M1) showed high levels of cell-mediated responses and showed less shedding than GFP controls (Boyd et al., 2013). A single-cycle “universal” influenza vaccine candidate based on the suppression of HA signal sequence (S-FLU) was shown to induce high levels of CD4+ and CD8+ T cell immune responses when administered to pigs by aerosol, which was correlated to reduced viral titers following challenge with a closely related H1N1pdm09 virus (Morgan et al., 2016).

Because cell-mediated immune responses do not prevent infection but rather contribute to accelerated clearance and restrict disease progression, CTL-inducing vaccines are a great aid to help reduce disease severity and mortality after heterologous infection. Hence, to reach their full potential as a “universal” vaccine they would be most efficient if used in complement to antibody-stimulating technologies. Interestingly, two phase I clinical trials using different T-cell inducing technologies in prime-boost or simultaneous immunizations with seasonal vaccine formulations resulted in stimulation of both T cell and antibody responses in the elderly (Antrobus et al., 2014; Atsmon et al., 2014).

### Universal vaccine platforms

**Figure 3:**
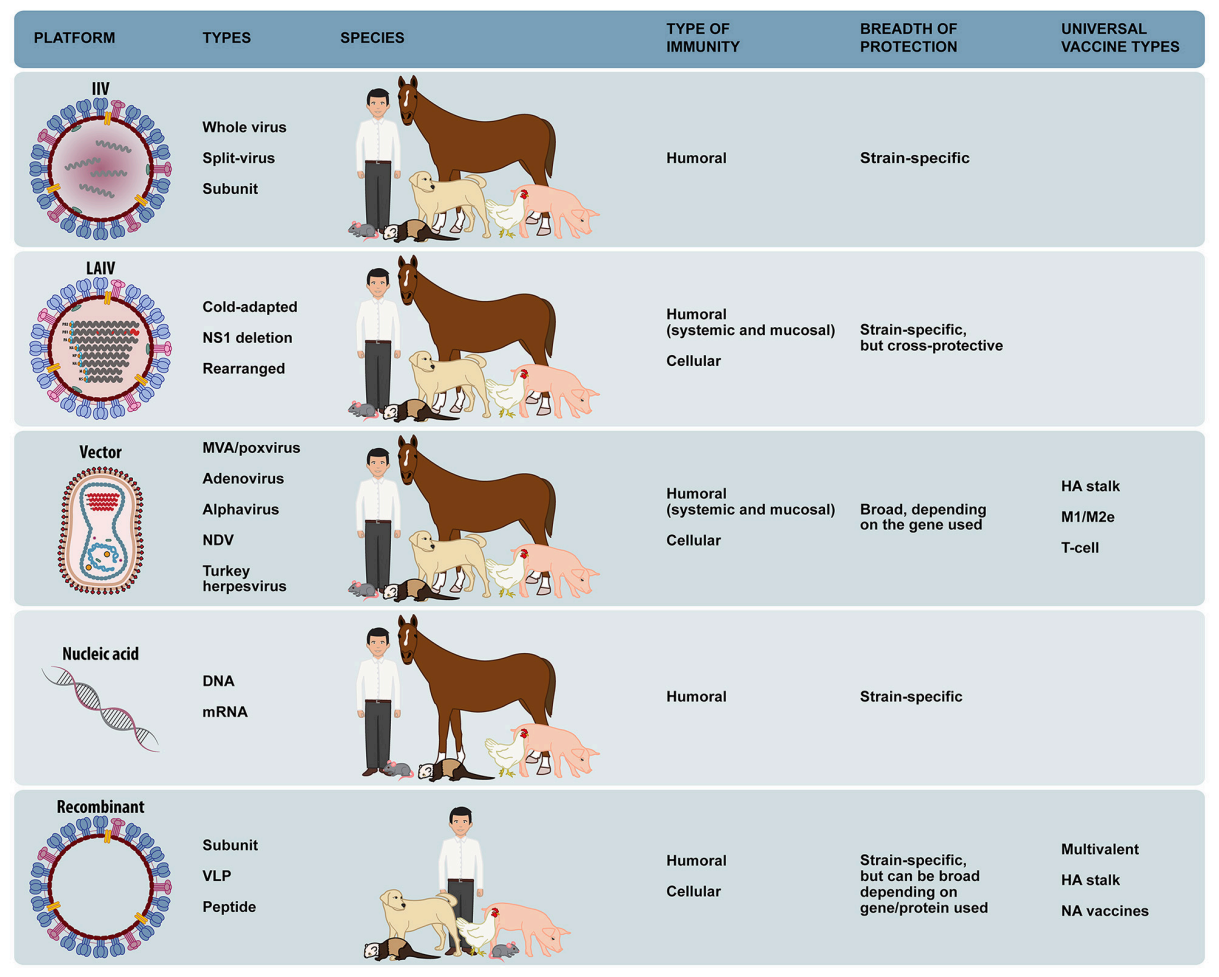
Influenza “universal” vaccine platforms that are used in multiple species or epidemiological situations. Vaccine platform, types of technology, host species, type of immunity stimulated, breadth of protection, and types of universally protective vaccines within each platform are shown.

In the effort to achieve better vaccine products, a myriad of vaccine manufacturing platforms and technologies have been developed with potential application in different species and different epidemiological situations. An ideal “universal” vaccine approach would include not only a broadly protective vaccine methodology, but also a more standardized vaccine platform that can be applied indistinctively in multiple animal species or in different epidemiological scenarios. Ideally, these “universal” approaches could be complementary to each other and could be used for either endemic, epidemic, and/or pandemic influenza. Some possible approaches that could serve as “universal” platforms are summarized in Figure 3 and will be discussed below.

#### Live-attenuated vaccines

As mentioned previously, cold-adapted/temperature sensitive LAIVs are already in use for humans and horses, and similar approaches have also been experimentally tested successfully in poultry and swine. In one approach, a combination of an HA tag at the C-terminus of PB1 along with temperature-sensitive mutations in PB1 and PB2 (tsHA LAIV) showed remarkable stability over multiple passages and in different IAV backbones of avian-, human-, and swine-origin. This strategy is safe and effective resulting in protection against low- and high-pathogenic influenza strains in chickens and against swine-origin IAVs in swine (Song et al., 2007; Pena et al., 2011). The tsHA LAIV has consistently shown cross protection after challenge with antigenically distinct viruses in swine and significantly more efficacious than inactivated products (Loving et al., 2013; Gauger et al., 2014). More recently, a prototypical influenza B virus engineered with a similar tsHA strategy resulted in a stable virus over multiple passages in tissue culture and eggs, and attenuated *in vivo*. In mice, a single intranasal dose of the IBV tsHA virus offered protection against lethal homologous and heterologous IBV challenges (Santos et al., 2017a).

Other strategies for the generation of attenuated virus have been described with great success in different species. NS1-truncated mutants can be used as LAIV candidates because they do not present the normal interferon-antagonist activity shown by viruses containing the wild-type NS1 protein (Richt and Garcia-Sastre, 2009). Several truncations of NS1 in both IAV and IBV strains resulted in virus attenuation and heterologous cross-protection (Solorzano et al., 2005; Hai et al., 2008; Pica et al., 2012). Additionally, this strategy has been proven to be immunogenic in young pigs, prime T cell response and result in partial heterosubtypic cross-protection (Kappes et al., 2012). In chickens, vaccination with a reassortant containing truncated-NS1 proteins harboring only 73 amino acids in addition to a modification in the HA to remove the polybasic cleavage site induced significant cross-protection against homologous and heterologous H5 clade viruses (Shi et al., 2016). Other modifications in the HA cleavage site can also result in attenuation of influenza viruses. By mutating one amino acid at the HA cleavage site, it is possible to engineer viruses carrying a cleavage site recognized by elastase instead of trypsin-like proteases, and these viruses are dependent on the presence of elastase to grow. Because elastase is not typically present in the respiratory tract, these viruses do not replicate efficiently *in vivo*. This technology has been tested and proven effective against homologous and heterologous challenge in swine (Masic et al., 2010).

Influenza viruses carrying reorganized or rearranged genomes serve as good LAIV candidates because they are attenuated *in vivo* and are highly unlikely to revert to a virulent phenotype. Particularly, the M and NS gene segments encode two polypeptides (M1 and M2 for M, NS1 and NEP for NS) by using splicing mechanisms (Paterson and Fodor, 2012; Wise et al., 2012), which allow for the manipulation and reorganization of the viral genome. An H9N2 virus was modified to express the H5 HA protein on the spliced NS gene segment, resulting in the expression of both H9 and H5 protein *in vitro* and protection of mice and ferrets against lethal H5N1 challenge (Pena et al., 2013). In a different approach, a reorganized virus was constructed by splitting the overlapping open reading frame (ORF) of the M segment and the resulting virus was shown to be attenuated *in vitro* and *in vivo* and protected mice from lethal homologous challenged (Nogales et al., 2016). More recently, an alternative rearranged design yielded a virus with two HA genes in the laboratory-adapted PR8 strain (Harding et al., 2017). The underlying goal of the work was to produce a platform that would overcome undesirable egg-adapted mutations that affect antigenicity of IAV, particularly of the H3 subtype. Modifications in segment 4 produced a segment carrying N1 NA and H1 HA genes from PR8 and a segment 6 carrying the H3 HA of a strain known to grow poorly in eggs and to mutate to facilitate growth. Alternative double HA viruses were produced carrying HA gene segments from current IAV and IBV vaccine strains. In general, double HA viruses grew to titers similar to the wt PR8 strain in eggs, carried no egg-adapted mutations and, as inactivated vaccines, produced protective anti-HA responses in mice against the corresponding HA genes (Harding et al., 2017). It remains to be determined whether such strategy would be amenable as an alternative LAIV in other animal species besides mice.

#### Virus vectors

Vectored vaccines (adenovirus, poxvirus, alphavirus, etc.) are a non-replicating safe approach for different species that eliminates the risk of virus recombination. Vector vaccines can mimic the natural infection when delivered through the intranasal route, and the antigens of interest are expressed in the native conformation, which results in higher antibody specificity. Additionally, this approach can be rapidly produced, is fully scalable and does not require the use of eggs. Not all virus vectors can be used in common for all species, but the concept of vectored-vaccine production tends to be similar between different vector systems. Modified vaccinia virus Ankara (MVA, poxvirus) is a highly attenuated strain of vaccinia virus that originated from growth selection on chicken embryo fibroblasts (CEF), and is one of the most commonly used vectors for human vaccines. Goodman et al. (2011) showed increased CD4+ and CD8+ T cell response in mice by using a prime/ boost regimen with DNA and MVA-vectors containing human T cell epitopes for M1, NS1, PB1, and PA proteins or conserved regions of H5N1 HA and NA on an NP backbone, which led to reduced viral replication and delayed mortality after challenge with H1N1 viruses. Similarly, MVA vectored vaccines expressing conserved proteins (HA stalk, HA stalk/M2e, M1, M2, PB1) of an H5N1 virus were not protective against lethal challenge in mice with H5N1, H7N1, or H9N2 virus; however adding the NP protein to the HA stem or HA stem/M2e resulted in cross-protection due to the induction of broadly-reactive virus-specific CD4+ and CD8+ T-cells (Hessel et al., 2014). MVA-based H1N1pdm09 vaccine fully protected macaques from a homologous challenge and an MVA vaccine encoding HA and NP from an H5N1 cross-protected animals against the H1N1pdm09 infection (Florek et al., 2014). MVA-based vaccines have also been tested in chickens: a MVA-H9 vaccine reduced clinical signs and virus shedding against homologous challenge, but did not prevent infection (Ducatez et al., 2016). Other systems using alphavirus, parainfluenza virus, or adenovirus have been developed as influenza vaccine vectors and pre-clinical studies have shown strong induction of anti-HA antibodies and T cell responses and some showed protection against homologous infection (Yang et al., 2009; Li et al., 2015; Wang et al., 2017).

Although replication-defective vectors can mediate potent protection against influenza virus infection, replication-competent vectors are considerably more potent since they can amplify transgene expression by several-fold. A single-cycle adenovirus (Ad) vector vaccine expressing PR8 HA resulted in markedly higher HA expression and HI titers in cotton rats than a non-replicating vector, and increased protection against homologous infection (Crosby et al., 2017). Another replication-defective vaccine using adenovirus serotype 5 (Ad5) has been tested in pigs and resulted in moderate protection against heterologous challenge (Wesley et al., 2004; Braucher et al., 2012). A single-cycle, propagation-defective alphavirus-like replicon particle vaccine, based on an attenuated Venezuelan equine encephalitis virus, was recently approved to be used in swine (United States Department of Agriculture, 2014). This technology allows for rapid strain updates and the ability to generate custom made vaccines with circulating strains (autogenous). This alphavirus-replicon vaccine induced robust immune response against H1 or H3 viruses in pigs, and completely protected pigs against homologous challenge and partially protected against heterosubtypic infection (Vander Veen et al., 2012, 2013). A propagation-defective alphavirus-like replicon particle vaccine expressing an H5 HA, with a similar approach to the one approved for use in swine, was shown to provide partial protection in turkeys against a HPAIV H5N2 strain with homologous HA (Santos et al., 2017b). Newcastle disease virus (NDV)-vectored H5 and H7 vaccines induced high levels of HI antibodies and protected chickens from challenge with a H7N9 or HPAI H5N1 viruses, respectively (Liu et al., 2015). Although NDV-vectored vaccines were shown to be effective in protecting chickens against HPAIV infection, preexisting immunity to the NDV vector limits the protective efficacy of these vectored vaccines in the field (Spackman and Pantin-Jackwood, 2014). An alternative chimeric NDV-vectored with F and HN ectodomains replaced by those of avian paramyxovirus serotype-2 was shown to be safe, not cross-react with NDV, and to partially protect 1-day-old immunized chickens against H5N1 HPAIV challenge (Kim et al., 2017). Additionally, a recombinant turkey herpesvirus vector vaccine expressing the HA gene of H5N1 HPAIV consistently demonstrated a high degree of homologous protection as well as cross-protection against heterologous clades of the H5N1 HPAIV (Gardin et al., 2016). One of the advantages of using recombinant vectored vaccines against avian influenza for poultry is the possibility of using automated methods as a spray or in drinking water for mass-immunization, providing a rapid, efficient, and economical immunization method.

#### Nucleic acid-based vaccines

Nucleic acid-based vaccines allow for the expression of target antigens *in vivo* and, therefore, induce both cellular and broad antibody immunity without exposing the host to a live virus. Hence, this technology provides a rapid, stable, highly scalable and safe alternative to traditional influenza vaccine production, without the need to grow virus in eggs (Kutzler and Weiner, 2008), and has been effectively tested in multiple host species. DNA vaccines against influenza are often based on expression of the HA protein, but the humoral immunogenicity is considered suboptimal in humans and large animals when compared to the traditional vaccine approaches. To increase their immunogenicity, further optimization has been proposed, including the use of adjuvants, different prime-boost combinations, or different delivery methods. In a recent study, by using the NK cell agonist α-Galactosylceramide as an adjuvant in mice, there was a significant increase in the IgG titers and IFN-γ levels compared with mice receiving the DNA vaccine alone (Fotouhi et al., 2017). Others have used the priming regimen to increase DNA vaccine immunogenicity. In a phase 1 trial in healthy adults, Crank and colleagues showed that a H1 DNA vaccine was safe but only modestly immunogenic as a single agent; however, antibody responses were significantly improved after boosting with a H1N1pdm09 monovalent inactivated vaccine (MIV) (Crank et al., 2015). Similarly, using a combined prime-boost regimen with DNA and protein vaccines significantly enhanced the humoral response of an H5 DNA vaccine in chickens (Stachyra et al., 2017). Alternative delivery systems, such as intradermal delivery, have been shown to improve levels of cross-reactive antibody and cell-mediated responses in pigs and humans (Ledgerwood et al., 2012; Borggren et al., 2016).

Messenger RNA (mRNA)-based vaccines, another variation of the nucleic acid-based technology, offers safety advantages in comparison to DNA-based vaccines since it harbors only components required for protein expression, is rapidly degraded and does not interact with the host genome. An intradermal mRNA vaccine encoding the full-length PR8 H1 protected young and old mice from lethal challenge with H1N1, H3N2, and H5N1 viruses, while the same mRNA vaccine encoding HA, NA and NP protected pigs from disease and reduced virus shedding after homologous challenge (Petsch et al., 2012). More recently, a modified mRNA vaccine encoding HA of H7N9 formulated with lipid nanoparticle generated robust immune responses in mice, ferrets, and nonhuman primates, protected mice from lethal H7N9 challenge and reduced lung virus titers in ferrets after homologous challenge (Bahl et al., 2017). The same vaccine platform encoding H10N8 HA was safe and immunogenic in humans in a randomized, double-blinded, placebo-controlled phase 1 trial (Bahl et al., 2017).

#### Protein-based and virus-like particle vaccines

Vaccines produced using insect cell and baculovirus expression vector systems (BEVS) allows for expression of large quantities of HA and/or NA *in vitro*, relatively fast manufacturing cycles, lack of emergence of spurious adaptive mutations as seen in other systems, and ability to produce the vaccine without the need for high-level biocontainment. A trivalent recombinant HA vaccine has been licensed for use in individuals 18–49 years of age (Flublok by Protein Sciences Corporation) and was shown to be safe and immunogenic in healthy adults and the elderly, but not in children (King et al., 2009; Baxter et al., 2011). Recombinant baculovirus-expressed vaccines have been tested in swine and poultry, and thus could be used as a universal platform. A recent study showed that three recombinant H1 constructs, either displayed in the baculovirus envelope, displayed in a Feline Leukemia Virus (FeLV) gag virus-like particle (VLP), or purified as a subunit IgG fusion protein, protected pigs against heterologous, homosubtypic H1N1 challenge (Hernandez et al., 2016). Furthermore, recombinant baculovirus-expressed H5 and H7 proteins administered with a water-in-oil adjuvant protected chickens against a lethal homosubtypic infection and reduced viral shedding in some animals (Crawford et al., 1999).

Virus-like particles (VLPs) are recombinant virus particles formed solely by viral structural proteins that do not have any genomic component. Although these are non-replicating particles, they retain the morphology of the virus and, therefore, the antigenicity and can activate innate immunity. VLPs can be produced in several expression systems, including baculovirus-, insect cell-, bacteria-, or plant-derived systems (Low et al., 2014; Pillet et al., 2016; Valero-Pacheco et al., 2016; Li et al., 2017). In a double-blind, randomized phase I clinical trial, Low and colleagues showed that a non-adjuvanted H1NIpdm09 VLP vaccine was safe and resulted in seroconversion in more than 50% of the subjects with just one dose, and this percentage increased after boost (Low et al., 2014). A recent cross-sectional study demonstrated that subjects previously vaccinated with a H1N1pdm09 HA VLP vaccine in a phase 2, randomized, double blind, placebo-controlled study still had detectable levels of antibodies 24 months after vaccination (Valero-Pacheco et al., 2016). VLPs also represent a promising approach for broadly cross-protective “universal” vaccines, as shown by a recent phase I-II randomized clinical trial in which a quadrivalent VLP vaccine induced significant HI titers against all components of the vaccine and also cross-reactive HI responses against heterologous strains (Pillet et al., 2016). This cross-reactive response was also observed in chickens vaccinated with triple-subtype (H5, H7, and H9) VLPs that were protected against challenge with heterologous HPAI H5N2 and H7N3 and LPAI H9N2 (Pushko et al., 2017). Vaccination of chickens with adjuvanted VLPs expressing H9N2 HA and NA also induced robust HI antibodies and reduced viral shedding after homologous challenge (Li et al., 2017). In pigs, VLP antigens consisting of H1N1pdm09 HA, NA, and M1 proteins elicited robust levels of humoral and mucosal immune responses and protected pigs against homologous infection (Pyo et al., 2012).

## Closing remarks

Current vaccines in use for humans and animals do not provide long-lasting broad protection. Novel strategies are available with the potential of providing “universal” broadly cross-protective and memory-stimulating immune responses against several or all influenza strains. Despite great progress in recent years, the “universal” vaccine technologies still present several obstacles on the path to licensing. To reach a full “universal” potential, vaccines should stimulate both antibody and T-cell immune responses to the more conserved epitopes of influenza viruses. One way to reach this full breadth of the immune system is to use live-attenuated vaccines or live vectored-vaccines to deliver these conserved proteins, while stimulating cellular and mucosal immunity. Questions remain regarding to the duration of the immunity of “universal” vaccines, how easily these strategies can be used in a field situation, if these vaccines will provide better protection than the current vaccines without the need for frequent updates, or if they should be used in combination with current technologies. More importantly, correlates of protection are not well defined for these novel technologies, and standardized assays to measure their protection levels need to be established.

Successful control of influenza can only be achieved through collaborative support between human and animal health. Any attempts in developing broadly cross-reactive vaccines should take under consideration the complex ecology of IAVs and how inter-related the viruses are between different species. Vaccine platforms that can be used indistinctively in various animal species and that can easily be accessed in different epidemiological scenarios (e.g., during a season, outbreak, or pandemic) could greatly improve vaccine production as such approach could create standardized regulations for vaccine licensing for different hosts and streamlined vaccine production processes. Such uniform approach is not necessarily novel. One could argue that production of inactivated vaccines follows similar paths irrespective of the targeted species. Thus, “universal vaccine platforms” for “universal vaccines” would ultimately be a great complement to drastically reduce the disease burden caused by influenza viruses in people and animals.

## Author contributions

DR and DP have contributed in the conceptualization of the ideas, drafting the manuscript, critical revision of the manuscript, and final approval.

### Conflict of interest statement

The authors declare that the research was conducted in the absence of any commercial or financial relationships that could be construed as a potential conflict of interest.
